# Papillary thyroid carcinoma in thyroglossal duct cyst: a Peruvian case series

**DOI:** 10.1530/EDM-25-0139

**Published:** 2026-01-08

**Authors:** José Luis Paz-Ibarra, Marialejandra Delgado Rojas, Edward Paucar Holgado, Jenyfer María Fuentes-Mendoza, Luis Concepción-Urteaga, Juan Eduardo Quiroz-Aldave, Marcio José Concepción-Zavaleta, José Somocurcio Peralta

**Affiliations:** ^1^ School of Medicine, Universidad Nacional Mayor de San Marcos, Lima, Perú; ^2^ Division of Endocrinology, Hospital Nacional Edgardo Rebagliati Martins, Lima, Perú; ^3^ Division of Endocrinology, Hospital Nacional Almanzor Aguinaga, Chiclayo, Perú; ^4^ Grupo de Investigación en Neurociencias, Metabolismo, Efectividad Clínica y Salud Pública, Universidad Científica del Sur, Lima, Perú; ^5^ School of Medicine, Universidad Nacional de Trujillo, Trujillo, Perú; ^6^ Division of Research, Hospital de Apoyo Chepén, Chepén, Perú; ^7^ Division of Pathology, Hospital Nacional Edgardo Rebagliati Martins, Lima, Perú

**Keywords:** papillary thyroid cancer, thyroid neoplasms, thyroglossal cyst, case reports

## Abstract

**Summary:**

Papillary thyroid carcinoma (PTC) is the most frequent type of differentiated thyroid cancer, while thyroglossal duct cysts (TGDCs) are common congenital anomalies of the neck. The coexistence of PTC within a TGDC is exceptionally rare, with a reported incidence of less than 1.5%. We present three Peruvian cases of PTC arising in TGDCs. The patients (two females and one male; age range: 34–47 years) presented with progressive midline cervical masses of two to four years’ duration. All underwent cervical ultrasound, contrast-enhanced computed tomography, and fine-needle aspiration biopsy. Histopathology confirmed PTC, including classical and follicular variants. Surgical management varied: two patients underwent Sistrunk procedure alone, while one required Sistrunk surgery followed by total thyroidectomy, cervical lymph node dissection, and radioactive iodine (RAI) therapy. All patients remain disease-free after 6–12 years of follow-up. The diagnosis of carcinoma in TGDC is often incidental, but preoperative imaging and cytology can raise suspicion. Optimal management remains controversial; while the Sistrunk procedure may be sufficient in most cases of PTC confined to TGDC without extracapsular extension, in some scenarios, such as the presence of suspicious thyroid nodules, extracystic extension, and cervical lymph node metastasis, an additional thyroidectomy and RAI therapy may be warranted. In conclusion, PTC in TGDC is a rare entity with generally favorable prognosis. Early recognition, individualized treatment, and multidisciplinary decision-making are essential for optimal outcomes.

**Learning points:**

- Suspect malignancy in any persistent or enlarging midline neck mass, especially when imaging shows solid components or calcifications.
- FNAB may have variable diagnostic yield in cystic lesions due to low cellularity; accuracy can be improved by aspirating cystic contents first or targeting the solid components of the cyst.
- Treat with the Sistrunk procedure, adding total thyroidectomy and/or radioiodine based on oncologic risk and nodal involvement.
- Expect a generally favorable prognosis, but maintain long-term surveillance given the (low) risk of recurrence.
- Guidelines regarding management of thyroglossal duct cyst cancer have recently been included within ATA 2025 thyroid cancer guidelines.

## Background

Differentiated thyroid carcinoma accounts for 95% of thyroid cancers and is classified into follicular thyroid carcinoma and papillary thyroid carcinoma (PTC). PTC is the most common type, representing approximately 80% of cases, with about 44,000 new diagnoses annually in the United States. Women are more frequently affected, with a female-to-male ratio of 2.5:1 (1, 2, 3). The main etiological factors include direct radiation exposure and familial genetic mutations, most notably in the *BRAF* (45%), *RAS* (0–20%), and *TERT* genes. Clinically, PTC often presents as asymptomatic thyroid nodules, although depending on size and location, patients may develop dysphagia, dysphonia, or dyspnea. The disease usually demonstrates slow growth and a favorable long-term prognosis, with mortality rates ranging from 0.2 to 0.4 per 100,000 in men and from 0.2 to 0.6 per 100,000 in women (4, 5, 6).

Conversely, the thyroglossal duct cyst (TGDC) is a developmental anomaly caused by the persistence of the thyroglossal duct, which normally involutes by the 10th week of gestation. TGDC is the most common congenital cervical anomaly in children, accounting for up to 70% of such cases, and occurs in approximately 7% of adults. It shows a female predominance with a 1.5:1 ratio compared to males. Ultrasonography is the initial diagnostic tool of choice due to its 90% accuracy and low cost, enabling localization of the cyst in the midline neck and assessment of the thyroid gland. Scintigraphy may also be useful for identifying ectopic thyroid tissue when the gland is absent from its usual location. Management is primarily surgical, involving cyst resection and partial removal of the hyoid bone through the Sistrunk procedure, with reported recurrence rates between 0 and 8% (7, 8).

The development of PTC within a TGDC is an exceptionally rare condition, with an incidence of less than 1.5%. It typically manifests around the age of 40 and is more common in women. Diagnosis is often incidental following Sistrunk procedure, when histological examination reveals PTC. Owing to its rarity, management remains controversial, with proposed strategies including total thyroidectomy, radioiodine therapy, and hormonal replacement. Furthermore, its pathogenesis is debated: some authors suggest that PTC originates primarily in the thyroid and metastasizes to the cyst, whereas others argue that the carcinoma arises within the cyst and subsequently spreads to the thyroid gland (9, 10, 11).

Although management recommendations for PTC arising in thyroglossal duct cysts were historically limited, with earlier studies suggesting broader surgical approaches and recent ATA 2025 guidelines providing initial direction (12, 13, 14), evidence remains scarce due to the rarity of this entity. Here, we present a case series reporting the coexistence of these entities, highlighting their rarity and the clinical approach undertaken.

## Case presentation

### Case 1

**Figure 1:**
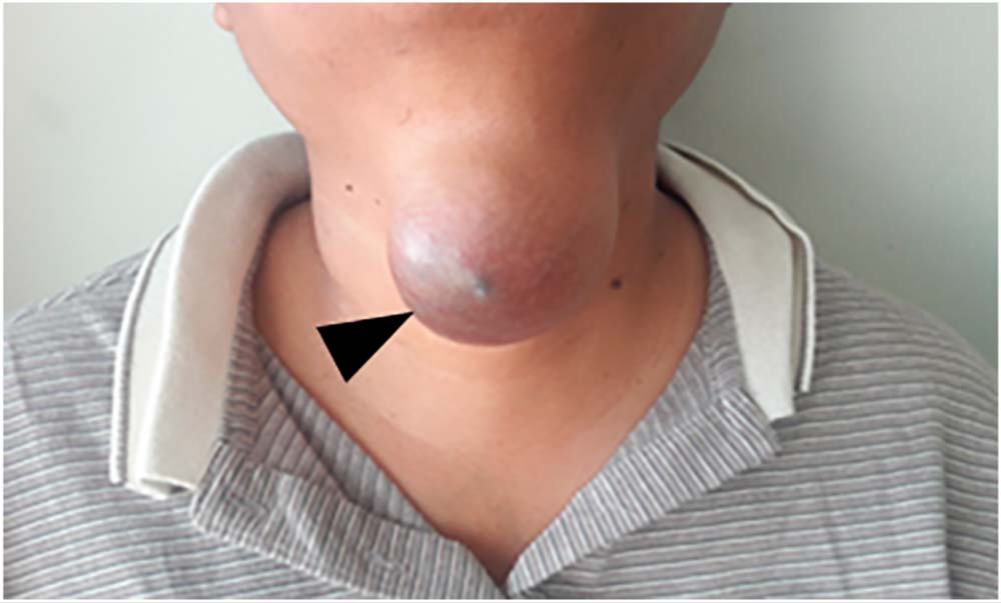
Clinical image of an anterior cervical mass.

Case 1 is a 34-year-old male with a 4-year history of progressive anterior cervical mass. On physical examination, a midline supra-hyoid mass measuring approximately 10 × 8 cm was palpated; it was non-tender and semi-solid, with skin discoloration but no signs of inflammation (Fig. 1).

### Case 2

Case 2 is a 35-year-old female with a 2-year history of progressive anterior cervical mass. Physical examination revealed a 5 × 3 cm midline submental mass, soft, non-tender, and without inflammatory signs.

### Case 3

A 47-year-old female with a history of left hemithyroidectomy for a 3 cm symptomatic thyroid nodule, 3 years back, previously reported as a colloid goiter, was referred for thyroid function evaluation. On physical examination, a 2.5 cm midline upper cervical mass was noted, firm and mobile with swallowing.

## Investigation

### Case 1

**Figure 2:**
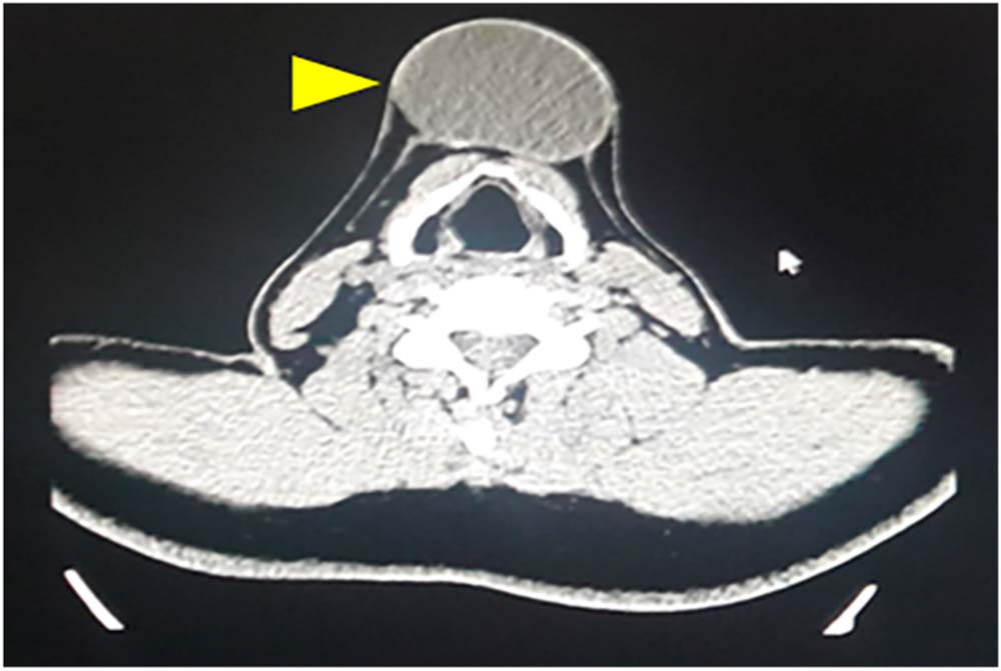
Contrast-enhanced CT scan of the neck showing an encapsulated cystic lesion.

Laboratory tests were unremarkable. Cervical ultrasound revealed a homogeneous thyroid gland of normal size and echogenicity, and in the right lobe, a well-defined hypoechoic nodule measuring 9 × 7 mm with soft consistency was observed on elastography. In the midline supra-hyoid region, a cystic lesion with sediment and coarse calcifications measuring 4.8 × 3.9 × 5.9 cm (volume 58.5 cc) was identified, without cervical lymphadenopathy. Neck CT with contrast showed a lobulated, encapsulated bilocular cystic lesion with small calcifications, measuring 33 × 30 × 15 mm, located in the cervical midline (Fig. 2). Ultrasound-guided FNAB of the midline cystic lesion revealed a Bethesda category V lesion, suspicious for malignancy. Cytopathology of the thyroid nodule was benign, Bethesda category II.

### Case 2

Ultrasound showed a mixed lesion measuring 46 × 24 mm, multiloculated, with a 17 × 9 mm solid component and multiple calcifications. Neck CT demonstrated multiloculated cystic image extending from the submental to the hyoid region, measuring 42 × 35 mm. FNAB with ultrasound guidance yielded atypical cells suspected of malignancy.

### Case 3

**Figure 3:**
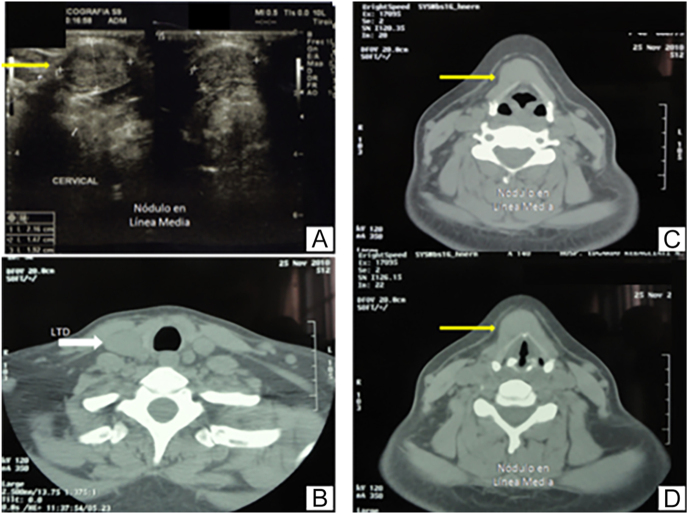
Cervical ultrasound showing a prehyoid nodule (A). Cervical CT demonstrating a homogeneous prehyoid nodule and the absence of nodules in the thyroid gland (B, C, D). In image (A), the arrow points to the cyst on the ultrasound; in image (B), the arrow points to the right lobe without nodules on the CT scan; and in images (C and D), the arrows point to the thyroglossal duct cyst on the CT scan.

Biochemical studies were normal. Cervical ultrasound showed a solid midline supra-hyoid nodule measuring 21 × 14 × 19 mm. Cervical CT revealed an oval pre-cartilaginous midline lesion measuring 18 mm (Fig. 3). FNAB confirmed papillary carcinoma.

## Treatment

### Case 1

The patient underwent Sistrunk procedure, where a 7 cm supra-hyoid mass with anterior wall necrosis, skin thinning, and a superficial prelaryngeal lymph node was found. Histopathology showed PTC, predominantly classical variant with a focal follicular variant, maximum tumor diameter 1 cm, with free surgical margins. The lesion infiltrated the capsule, the adjacent bone, and lymphatic vessels, while perithyroidal and submental lymph nodes were negative for malignancy.

### Case 2

**Figure 4:**
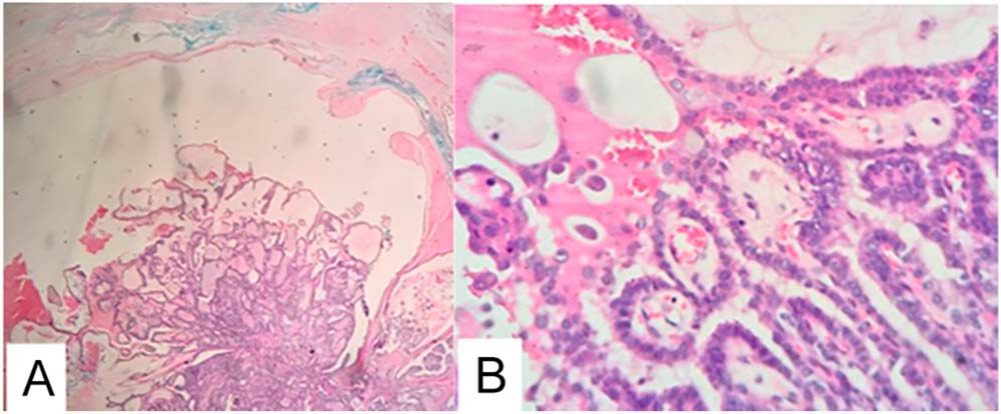
Panoramic microphotograph with hematoxylin–eosin (H&E), 10×: intracystic papillary tumor lesion; cyst wall composed of fibroconnective tissue without evidence of peripheral thyroid tissue (A). Microphotograph with H&E, 40×: intracystic papillary tumor composed of cells with papillary arrangement and characteristic nuclear chromatin (B).

**Figure 5:**
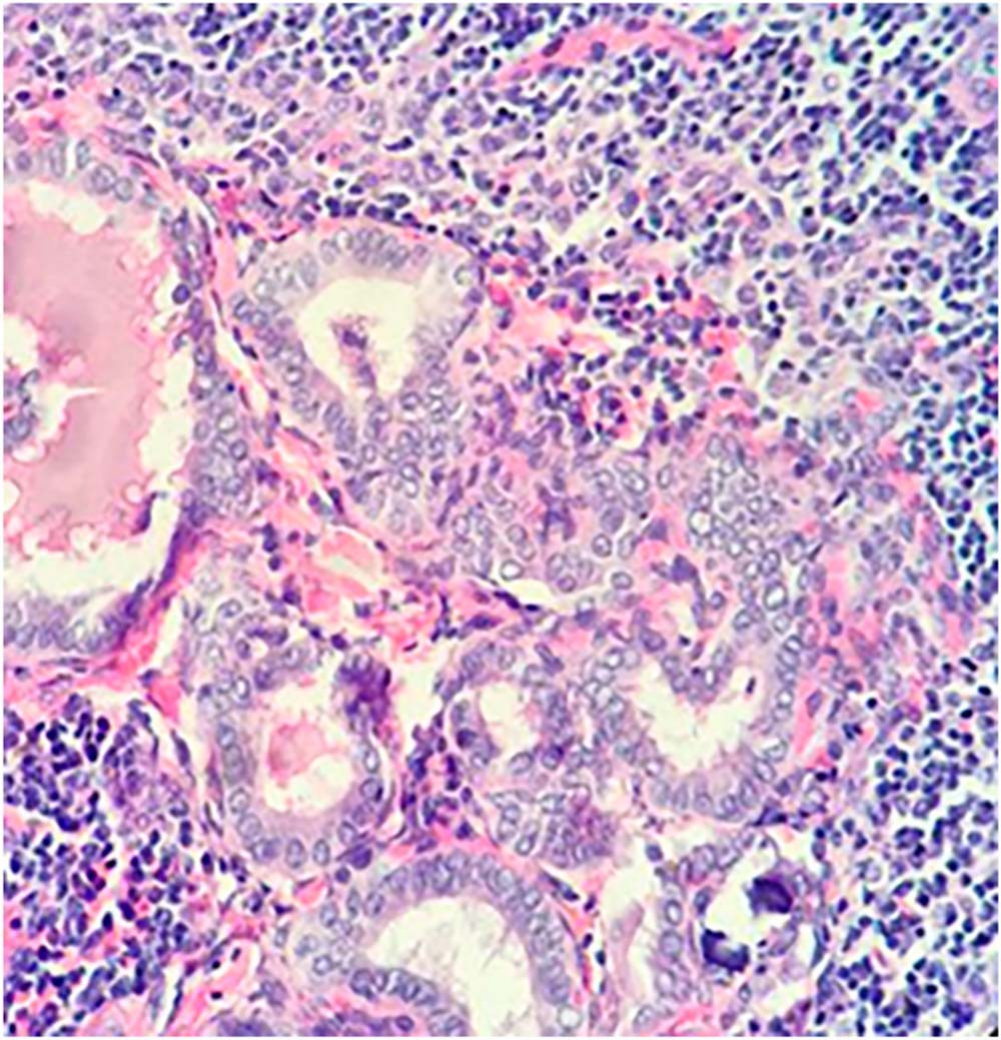
Microphotograph with H&E, 40×: cervical lymph node showing a metastatic tumor nest with nuclear features of papillary carcinoma.

The patient underwent Sistrunk procedure, during which a 40 × 40 mm tumor adherent to the hyoid bone and infrahyoid muscles was resected. Based on the positive frozen section for PTC within the TGDC, the surgical team proceeded with a total thyroidectomy and central cervical lymph node dissection. Histopathological analysis confirmed PTC arising from the TGDC with cyst wall infiltration (Fig. 4) and involvement of one lymph node (Fig. 5). Examination of the thyroid revealed a 6 × 4 × 3 mm sclerosing PTC in the right lobe, without evidence of capsular invasion. The patient subsequently received ablative radioactive iodine (RAI) therapy.

### Case 3

**Figure 6:**
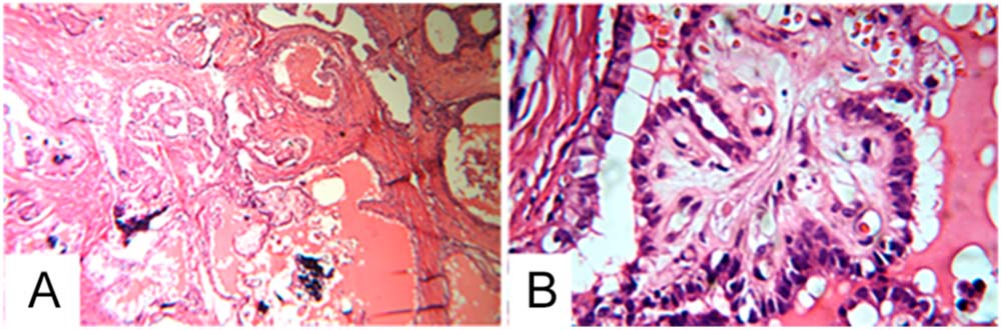
Microphotograph with H&E, 10×: intracystic papillary tumor composed of papillary-arranged cells (A). Microphotograph with H&E, 40×: epithelial proliferation with papillary structures and atypical nuclei, typical features of PTC (B).

Sistrunk procedure was performed, revealing a nodule with an intralesional cystic cavity filled with brownish tumor tissue. Histopathology demonstrated a 2.5 × 2 cm papillary carcinoma, predominantly follicular variant (80%) with classical component (20%), infiltrating the cyst wall and with lymphovascular emboli (Fig. 6). Completion thyroidectomy was not performed because the patient did not give authorization.

In all three cases, the pathology confirmed the existence of a cyst remnant with epithelial lining and ectopic thyroid tissue, ruling out the possibility of cystic lymph node metastasis.

Table 1 summarizes the demographic data, diagnostic methods, and treatments for the reported cases.

**Table 1:** Main characteristics of the reported cases.

Characteristics	Case 1	Case 2	Case 3
Gender	Male	Female	Female
Age (years)	34	35	47
Diagnostic methods			
Cervical ultrasound	Yes	Yes	Yes
Contrast-enhanced cervical CT	Yes	Yes	Yes
FNAB of cervical lesion	Yes	Yes	Yes
Histopathology			
Confirmed existence of a cyst remnant with epithelial lining and ectopic thyroid tissue	PTC, classical and follicular variants	PTC, classical variant	PTC, classical and follicular variants
Procedures	Sistrunk procedure	Sistrunk procedure + total thyroidectomy with central cervical lymph node dissection + RAI therapy	Sistrunk procedure
Current status	NED, 6 years	NED, 8 years	NED, 12 years

CT, computed tomography; FNAB, fine-needle aspiration biopsy; RAI, radioactive iodine; PTC, papillary thyroid carcinoma; NED, no evidence of disease.

## Outcome and follow-up

### Case 1

The patient recovered well postoperatively and remains under endocrinology follow-up without evidence of disease. He is receiving levothyroxine (LT4) replacement therapy to maintain TSH < 2 mIU/L.

### Case 2

The patient remains asymptomatic and disease-free under endocrinology follow-up. She is currently receiving LT4 replacement therapy to maintain TSH < 2 mIU/L.

### Case 3

The patient continues regular endocrinology follow-up without evidence of recurrence. She is receiving levothyroxine (LT4) replacement therapy to maintain TSH < 2 mIU/L.

## Discussion

The TGDC is the most common cervical anomaly, with a prevalence of 7% in adults and up to 70% in children (7, 15). The literature indicates that the most frequent location of TGDC is at the level of the hyoid bone (61%), followed by the supra-hyoid region (24%), the sternum (13%), and the tongue (2%) (16).

PTC accounts for approximately 80% of all thyroid cancers (17). However, the presence of carcinoma within a TGDC is rare, with a prevalence of less than 1.5% (9, 10, 11, 18). One study reported 4 cases of PTC among 90 patients with TGDC, corresponding to an incidence of 4.4% (19). Similarly, another study described 22 carcinomas arising from TGDCs in a cohort of 685 patients, with an incidence of 3.2% (20).

Carcinoma of the TGDC typically lacks specific symptoms, and clinical manifestations largely depend on tumor size and location (21). Patients often present due to tracheal compression caused by rapid tumor growth, in addition to the characteristic physical findings of a TGDC. In most cases, the sole clinical feature is a neck swelling without additional symptoms (22). Nevertheless, pain, voice changes, rapid enlargement of the mass, weight loss, lymphadenopathy, and respiratory symptoms such as airway compression may suggest malignant transformation. These features, however, are uncommon and generally associated with poorer prognosis (23). In our three reported cases, patients presented with midline cervical masses without compressive symptoms, consistent with the usual presentation and overall favorable prognosis.

The diagnosis of TGDC carcinoma is confirmed histopathologically, where hyperchromatic nuclei and intracytoplasmic inclusion bodies are diagnostic hallmarks (24). Thyroid ultrasound, cervical CT, thyroid function tests, and FNAB are the main diagnostic tools, with FNAB considered the gold standard. In cysts with abundant fluid, aspiration prior to biopsy is recommended. The BRAF V600E mutation is associated with malignancy and possible lymph node metastasis. Ultrasound is also useful to assess cyst extension and identify suspicious findings, such as solid nodules, calcifications, or wall thickening, with the “claw sign” described as a characteristic feature (25). In this case series, ultrasound was the initial diagnostic modality, identifying calcifications in most cases. Contrast-enhanced CT further helped delineate cyst size and the extent and relationship of the injury to adjacent organs and exclude lymph node involvement. FNAB confirmed carcinoma in all three cases.

The rarity of this condition precluded the development of standardized management guidelines at the time our patients were treated. Consequently, therapeutic decisions were made by our multidisciplinary team based on available evidence from case reports, series, and institutional experience, which explains the variation in our surgical approach.

In our series, this individualized approach was applied as follows: cases 1 and 3, who had disease without concomitant thyroid gland involvement, despite showing microscopic local infiltration limited to adjacent soft tissues, underwent Sistrunk procedure alone and achieved favorable long-term outcomes.

During the time our patients were treated, there were no recommendations for the management of PTC in TGDC, so decisions were made by consensus in our multidisciplinary DTC group. Some studies proposed the use of some markers or the presence of some characteristics to opt for a surgery beyond the Sistrunk procedure: an Italian group in 19 patients found that omitting total thyroidectomy in T1 thyroglossal cyst cancers or in cases with sonographically normal thyroid was associated with a 43% risk of missing a thyroid malignancy, and they concluded that routine addition of total thyroidectomy to the Sistrunk procedure appears appropriate for comprehensive locoregional surveillance, especially because selecting a subset of patients in whom it can be omitted is a difficult task (12). The same group demonstrated that BRAF V600E positivity appears to predict locally advanced disease requiring RAI therapy; therefore, it could serve as a preoperative tool to predict the need for total thyroidectomy in addition to the Sistrunk procedure (13). Recently, in the latest version of the 2025 ATA thyroid cancer guidelines, there are two recommendations for the management of this rare association (14).

The cornerstone of surgical management for these cases is the Sistrunk procedure, consisting of resection of the cyst, the tumor, the hyoid bone, and the surrounding central tissue along the thyroglossal tract (26). Adjuvant RAI therapy may be considered in selected clinical scenarios (27). The need for individualized decision-making has recently been addressed by the 2025 ATA guidelines (14), which now provide specific recommendations. They suggest that the Sistrunk procedure alone is sufficient for uncomplicated TGDC carcinoma. However, the addition of thyroidectomy (initial or completion) is recommended in cases with a suspicious thyroid nodule, large tumors in older patients, local extension, metastasis, or involvement of the Delphian lymph node.

The prognosis of TGDC carcinoma is generally favorable, except in the presence of the BRAF V600E mutation (28), which could not be evaluated in our setting. Among the histologic variants, squamous cell carcinoma is the most aggressive (29). The favorable postoperative course in our series supports the low recurrence and mortality rates associated with this neoplasm. Nonetheless, a periodic follow-up is essential to enable early detection of recurrence.

When comparing our findings with previously published case series, some similarities and differences become evident. As in prior reports, the majority of our patients were middle-aged adults with a slight female predominance and presented with asymptomatic midline cervical masses. Consistent with the literature, ultrasound and FNAB were instrumental in raising suspicion for malignancy, although the final diagnosis continued to rely on histopathological confirmation. However, our series also exhibited notable differences: i) all diagnoses were established preoperatively through FNAB rather than incidentally after Sistrunk procedure; ii) two cases demonstrated marked local infiltration of adjacent tissues, a feature not consistently described in previous reports; and iii) our long-term follow-up of 6–12 years exceeds that of many published series, providing additional confirmation of favorable outcomes.

This case series highlights the challenges in diagnosing and managing PTC arising in a thyroglossal duct cyst, a rare entity with an incidence of less than 1.5%. While diagnosis is usually incidental following Sistrunk procedure, in our series, it was established preoperatively. Management requires an individualized approach, which may include total thyroidectomy and radioiodine therapy depending on the patient’s oncologic risk. Despite the absence of consensus in the years that we treated our patients on the optimal therapeutic strategy, favorable outcomes with very low recurrence rates were achieved in most cases as in our Peruvian case series. These findings emphasize the importance of a multidisciplinary approach in therapeutic decision-making and carry implications for future management strategies.

## Declaration of interest

The authors declare no conflicts of interest prejudicing the impartiality of the research reported. MJ Concepción-Zavaleta is a Senior Editor of *Endocrinology, Diabetes & Metabolism Case Reports* and was not involved in the review or editorial process for this paper, on which he is listed as an author.

## Funding

This research did not receive any specific grant from any funding agency in the public, commercial, or not-for-profit sector.

## Patient consent

Written informed consent for the publication of their clinical details and clinical images was obtained from each patient.

## Author contribution statement

JP-I: conceptualization, resources, investigation, writing – review and editing. MDR: investigation, writing – original draft. EPH: investigation, writing – original draft. JMF-M: investigation, writing – original draft. LAC-U: investigation, writing – review and editing, project administration. JEQ-A: investigation, writing – original draft, writing – review and editing. MC-Z: investigation, writing – review and editing, project administration. JSP: investigation, writing – original draft.
